# FH535 Suppresses Osteosarcoma Growth *In Vitro* and Inhibits Wnt Signaling through Tankyrases

**DOI:** 10.3389/fphar.2017.00285

**Published:** 2017-05-23

**Authors:** Carl T. Gustafson, Tewodros Mamo, Kristen L. Shogren, Avudaiappan Maran, Michael J. Yaszemski

**Affiliations:** ^1^Department of Molecular Pharmacology and Experimental Therapeutics, Mayo Clinic College of Medicine, Mayo Clinic, RochesterMN, United States; ^2^Department of Orthopedic Surgery, Mayo Clinic College of Medicine, Mayo Clinic, RochesterMN, United States; ^3^Department of Physiology and Biomedical Engineering, Mayo Clinic College of Medicine, Mayo Clinic, RochesterMN, United States

**Keywords:** osteosarcoma, tankyrase inhibitor, PARP1, Wnt signaling, small molecule chemotherapy

## Abstract

Osteosarcoma (OS) is an aggressive primary bone tumor which exhibits aberrantly activated Wnt signaling. The canonical Wnt signaling cascade has been shown to drive cancer progression and metastasis through the activation of β-catenin. Hence, small molecule inhibitors of Wnt targets are being explored as primary or adjuvant chemotherapy. In this study, we have investigated the ability of FH535, an antagonist of Wnt signaling, to inhibit the growth of OS cells. We found that FH535 was cytotoxic in all OS cell lines which were tested (143b, U2OS, SaOS-2, HOS, K7M2) but well tolerated by normal human osteoblast cells. Additionally, we have developed an *in vitro* model of doxorubicin-resistant OS and found that these cells were highly responsive to FH535 treatment. Our analysis provided evidence that FH535 strongly inhibited markers of canonical Wnt signaling. In addition, our findings demonstrate a reduction in PAR-modification of Axin2 indicating inhibition of the tankyrase 1/2 enzymes. Moreover, we observed inhibition of auto-modification of PARP1 in the presence of FH535, indicating inhibition of PARP1 enzymatic activity. These data provide evidence that FH535 acts through the tankyrase 1/2 enzymes to suppress Wnt signaling and could be explored as a potent chemotherapeutic agent for the control of OS.

## Introduction

Osteosarcoma (OS) is the most common primary bone tumor, with approximately 1000 new cases in the United States every year (Fletcher et al., 2002). Ninety percent of OS patients are children and young adults between the ages of 10 and 30 (Ritter and Bielack, 2010). Lack of response to the standard chemotherapy regimen is the major cause for disease progression in OS (Bacci et al., 1993; Fletcher et al., 2002). Canonical Wnt signaling has been frequently tied to chemotherapy resistance and poor prognosis in OS; moreover, the current literature supports the concept that Wnt signaling is involved in tumor metastasis and proliferation in OS (Flores et al., 2012; Chen et al., 2015). Additionally, overexpression of Wnt promoting factors, and under-expression of endogenous Wnt inhibitors, are correlated with disease intensity and poor prognosis (Mandal et al., 2007; Lu et al., 2012). Still, the chemotherapy regimen in OS has remained constant in the past several decades, and newly developed Wnt-targeting chemotherapeutics have not yet reached approval for clinical use in cancers.

Activation of β-catenin by canonical Wnt signaling affects cell cycle progression, cellular differentiation, and susceptibility to chemotherapeutic agents (Pinto and Clevers, 2005; Basu et al., 2016; Nussinov et al., 2016). The tankyrase enzymes (TNKS1/2, also referred to as PARP5A/B), have emerged as attractive regulators of Wnt, due to the discovery of their role in Axin2 post-translational modification (Huang et al., 2009). The TNKS1/2 proteins are members of the poly-(adenosine diphosphate–ribose) polymerase (PARP) family of enzymes, and they serve to modify Axin2 by Poly(Adenosine diphosphate–Ribosyl)ation (PARylation), targeting Axin2 for destruction (Smith et al., 1998; Huang et al., 2009). Axin2 scaffolds a group of Wnt regulating proteins known as the β-catenin destruction complex, which poly-phosphorylates β-catenin. This phosphorylation targets β-catenin for ubiquitination and proteasomal destruction (Stamos and Weis, 2013). Decreased available Axin2 protein reduces elimination of β-catenin, allowing β-catenin to translocate to the nucleus where it stimulates the transcription of various target genes.

FH535 is a small molecule inhibitor of canonical Wnt signaling through β-catenin, and a number of reports have tested its utility for blocking Wnt signals (Handeli and Simon, 2008; Su et al., 2015; Wu et al., 2015). While FH535 has been shown repeatedly to inhibit Wnt signaling, the specific mechanism by which FH535 acts upon Wnt is yet unclear. We found that FH535 is indeed a potent inhibitor of Wnt signaling in OS, and is cytotoxic to the OS cell lines which were tested. Further experiments revealed that treatment with FH535 decreased PARylation of Axin2, as well as PARP1 auto-PARylation, indicating that FH535 acts on Wnt signaling through inhibition of the TNKS1/2 enzymes. These findings provide a conclusive mechanism for FH535 inhibition of canonical Wnt signaling, and suggest that non-specific blockade of PARP1 may confound Wnt-specific claims based on the effects of FH535. Moreover, this report provides preliminary data suggesting the utility of tankyrase inhibition in OS, and reveals critical information regarding a widely used inhibitor of Wnt signaling.

## Materials and Methods

### Mammalian Cell Culture

Human OS cell lines 143b, 143b-DxR, U2OS, SaOS-2, HOS, as well as the mouse OS cell line K7M2 were cultured in 75 cm^-2^ flasks in penicillin/streptomycin free Dulbecco’s Advanced Modified Eagles Media –F12 (DMEM-F12) formulation. The 143b-DxR cell line was derived from the 143b-wt cell line by repeated cycles of doxorubicin challenge, selection of resistant colonies, and expansion. Primary human osteoblast cells (HOBs) were obtained from cancellous bone surgical waste and established as cultured explants in accordance with procedures approved by the Mayo Clinic Institutional Review Board (IRB) and as previously described Robey and Termine (1985) and Wimbauer et al. (2012). In accordance with HIPAA and Mayo Clinic IRB authorization a waiver was obtained, and hence informed consent was not obtained prior to use of HOBs from surgical waste. Cells were stored in liquid phase nitrogen prior to use. Cell cultures were passaged prior to confluency and to a total of less than 10 passages before final use.

### Cytotoxicity Assay

Cells were plated at a density of 4 × 10^4^ cells per well in 24-well polystyrene dishes 16 h prior to treatment. Drug was diluted in serum free/penicillin free/streptomycin free DMEM-F12 and incubated for the desired time at 37°C. After treatment time, media was removed, wells were rinsed with Dulbecco’s phosphate buffered saline (DPBS), and cell viability was measured by Cell Titer 96^®^ MTS reagent assay (ProMega).

### Colony Forming Assay

Cells were cultured to 60–80% confluency as described above, trypsinized and counted using a hemocytometer. Cells were diluted in DMEM-F12, plated at desired concentrations in 6-well polystyrene dishes, and allowed to settle and attach to the plate for 4 h at 37°C. After settling, treatments were added to the dishes. Cells were incubated at 37°C for 7–12 days until countable colonies had formed. Colonies were stained with crystal violet and counted. Groups of >50 cells were defined as a colony.

### Protein Collection

Cells were plated at 5 × 10^5^ cells per dish in 100 mm dishes 16 h prior to treatment. Drug was diluted in serum free/penicillin free/streptomycin free DMEM-F12 and incubated for the desired time at 37°C. After treatment, dishes were washed with DPBS, and cells were scraped from the dishes into protease/phosphatase inhibitor solution containing complete mini protease inhibitor tablet (EDTA-free, Roche), 10 mM β-glycerophosphate, and 100 μM sodium orthovanadate. Collected cells were centrifuged briefly at 1,500 RPM, lysed, and centrifuged at 14,000 RPM for 10 min to pellet nuclear sediment and debris. Supernatant was collected and protein concentration was measured using Bio-Rad Protein Assay Reagent Dye (Bio-Rad).

### Western Blotting

Twenty microgram of protein was prepared with protein loading dye containing reducing agent and boiled for 3 min. Protein samples were separated by SDS-PAGE on 10% Tris-HCl protein separation gels, and transferred to Immobilon PSQ 0.2 μm PVDF membranes (Millipore). Membranes were blocked in TBST containing 5% milk, and incubated overnight at 4°C with primary antibody diluted in TBST containing 1% milk. Primary antibodies used: anti-GAPDH (1:5000, CST, D16H11), anti-PARP1 (1:1000, CST, 46D11), anti-Axin2 (1:400, Avivasysbio, OAAB02831). The following day, membranes were washed, incubated for 1 h at 20°C with goat anti-rabbit secondary antibody (1:2000, GE) diluted in TBST containing 1% milk, washed, and imaged on a ChemiDoc^TM^ Touch system (Bio-Rad).

### Immunoprecipitation

PAR modified proteins were immunoprecipitated according to described methods (Gagne et al., 2011). Tannic acid (200 μM) was included in wash solutions and cell lysis buffer as a PAR-glycohydrolase inhibitor. pADPr mouse monoclonal antibody clone 10H, anti-mouse IgG, as well as Protein G beads were purchased from Santa Cruz Biotechnology. The cell lysis buffer described by Gagne, et al. was prepared with the substitution of 20 mM pH 8.0 Tris-EDTA and 0.1% IGEPAL. Following immunoprecipitation, proteins were separated by SDS-PAGE on a 7.5% Tris-HCl gel (Bio-Rad). Membrane transfer, blocking, and antibody staining were performed according to standard western blotting methods.

### mRNA Transcript Analyses

RNA was collected and purified from treated cells using Trizol reagent (Invitrogen), and Zymo Quick-RNA^TM^ MicroPrep kit. 2.0 μg of RNA was used to produce cDNA using the High-Capacity cDNA Reverse Transcription Kit (Applied Biosystems, Fisher Scientific). qRT-PCR was performed on an ABI HT7000 Thermocycler using SYBR GreenER (Thermo-Fisher). A total of 10 μL reaction volume containing 25 ng of sample cDNA was used for each reaction. Primer sequences used: GAPDH F 5′-ATGTTCGTCATGGGTGTGAA-3′, R 5′-TGTGGTCATGAGTCCTTCCA-3′; Axin2 F 5′-AGAAATG CATCGCAGTGTGAAG-3′, R 5′-GGGTTCTCGGGAAATGA GGTA-3′; c-MYC F 5′-CGGATTCTCTGCTCTCCTCGAC-3′, R 5′-CCTCCAGCAGAAGGTGATCCA-3′; TNKS1 F 5′-TGGT GCTGATGTTCATGCAAA-3′, R 5′-ACAAGCTCCATGCTTTA GTAGC-3′; TNKS2 F 5′-GTGGCAATTCACTCCTCTTCA-3′, R 5′-TGGTGTGGGAGCCAAGTCTA-3′.

### Luciferase Activity

Cells were plated in 12-well dishes at 8 × 10^4^ cells per well. Sixteen hours after plating, cells were transfected with Super 8x Topflash (0.04 μg/well) and β-catenin (0.08 μg/well) using the FuGene 6 transfection reagent (Promega). The transfected cells were treated 24 h post-transfection for 48 h. After completion of the treatment, media was removed, cells were rinsed with DPBS, lysates were collected in Passive Lysis Buffer (Promega) according to manufacturer’s instructions and luciferase activity was measured using single Luciferase Assay System (Promega).

### Cell Cycle Analysis

143b-wt or 143b-DxR cells were seeded into 6-well polystyrene plates at 1.5 × 10^5^ cells per well. Sixteen hours after plating, cells were treated with vector (DMSO) or treatment groups for 24 h. After treatment, cells were harvested by trypsinization, then washed with and re-suspended in ice-cold PBS. After pelleting the cells by centrifugation (1,500 RPM for 5 min) equal volumes of PBS and 95% ethanol were added to the cells and stored at 4°C until further processing. RNase A (1.5 mg/ml; Sigma–Aldrich, St. Louis, MO, United States) was added to each tube before incubating them at 37°C for 15 min. Finally, propidium iodide (100 μg/ml; Roche, Indianapolis, IN, United States) was added to each tube prior to analysis by flow cytometry (Flow Cytometry Core at the Mayo Clinic).

## Results

### FH535 Is Highly Toxic in Osteosarcoma Cells, Does Not Affect Human Osteoblasts

**FIGURE 1:**
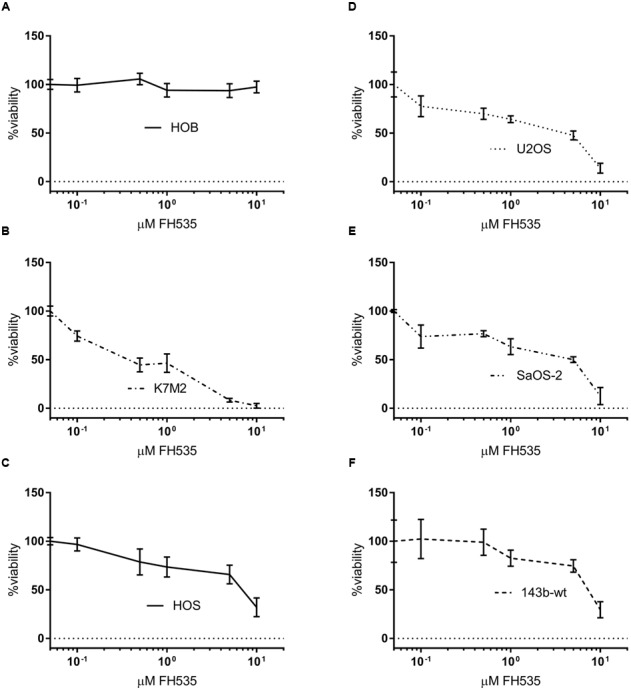
**FH535 is not toxic in human osteoblasts, but inhibits cell line viability in osteosarcoma cell lines (A–F)**. Viability of human osteoblasts **(A)** or osteosarcoma cell lines after 48 h treatment with FH535. (*n* = 3). Data information: in **(A–F)**, data are presented as mean, ±SD (*n* = 3 for all groups).

In all OS cell lines tested, we found that treatment with FH535 was highly toxic to the OS cells (**Figure 1**). This observation was in contrast to an observed lack of toxicity in cultured HOBs (**Figure 1A**). FH535 inhibited cell viability, as measured by MTS assay, decreased colony forming ability, and increased numbers of dead cells (Live/Dead staining) among OS cell lines at varying concentrations (**Figure 1** and Supplementary Figure 1). The U2OS (human) and K7M2 (mouse) OS cell lines exhibited increased sensitivity to FH535 treatment (**Figures 1B,D**), compared to other cell lines tested (143b-wt, SaOS-2, and HOS).

**FIGURE 2:**
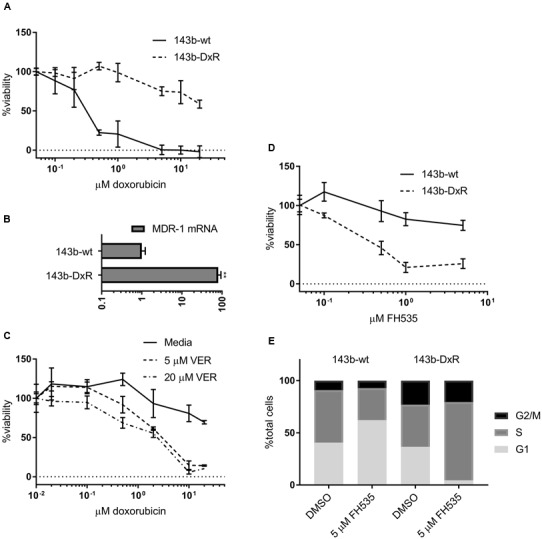
**Development and characterization of doxorubicin resistant osteosarcoma cell line (A).** Viability of 143b-wt and 143b-DxR cell lines treated for 48 h with doxorubicin. (*n* = 3). **(B)** Quantification of MDR-1 mRNA expression in 143b-wt and 143b-DxR cell lines. (*n* = 3). **(C)** 143b-DxR cell line treated with doxorubicin and verapamil or vector. Viability assessed at 48 h. (*n* = 3). **(D)** Viability of 143b-wt and 143b-DxR cell lines treated with FH535 for 48 h. (*n* = 3). **(E)** Cell cycle analysis of 143b-wt and 143b-DxR cell lines after treatment with FH535 for 24 h. Data information: in **(A–D)**, data are presented as mean, ±SD. **(B)** Statistical significance determined by unpaired *t*-test with Welch’s correction, ^∗∗^ indicates *p* < 0.01.

Additionally, we developed an *in vitro* cellular model of acquired doxorubicin resistance in OS in order to test the efficacy of Wnt inhibition in OSs that does not respond to the standard chemotherapeutic regimen. The resulting cells exhibited a high degree of resistance to doxorubicin compared to their parental line (**Figure 2A**) and overexpressed the ATP-binding cassette transporter family member Multidrug Resistance Protein 1 (MDR-1) (**Figure 2B**). These doxorubicin-resistant cells (143b-DxR) were able to be sensitized to doxorubicin by verapamil, which is a competitive inhibitor of MDR-1 (**Figure 2C**) (Safa, 1988). Thus, the 143b-DxR cell line developed a mechanism of resistance which is particularly dependent on MDR-1, a substrate of β-catenin mediated transcription (Lim et al., 2008; Flahaut et al., 2009; Correa et al., 2012). The viability data showed that the 143b-DxR cell line was highly sensitive to FH535 treatment, relative to its parental cell line (**Figure 2D**). Additionally, cell cycle analysis demonstrated G1 accumulation in the parental 143b-wt cells which had been treated with FH535, while the 143b-DxR cells accumulated in S-phase – demonstrating a response which was unique from the parental cell line, and more robust (**Figure 2E**).

### Topflash Luciferase Reporter and Axin2 mRNA Are Inhibited by FH535, While Axin2 Protein Is Increased

**FIGURE 3:**
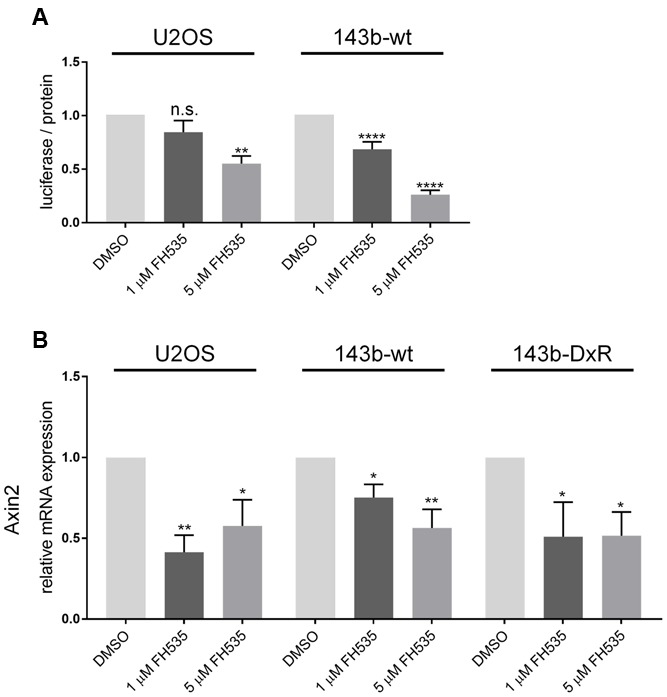
**Topflash luciferase activity and Axin2 mRNA indicate inhibition of Wnt signaling (A)**. Luciferase activity of Topflash reporter-transfected 143b-wt (left) and U2OS (right) cell line following 24 h treatment with FH535. (*n* = 6). **(B)** Expression of Axin2 mRNA after 24 h treatment with FH535 in 143b-wt, U2OS, and 143b-DxR cell lines. (*n* = 3). Data information: Data are presented as mean, ±SD. Statistical significance determined by one-way ANOVA with Tukey’s multiple comparison test, ^∗∗^indicates *p* < 0.01, ^∗∗∗∗^ indicates *p* < 0.0001, “n.s.” indicates no statistically significant difference.

**FIGURE 4:**
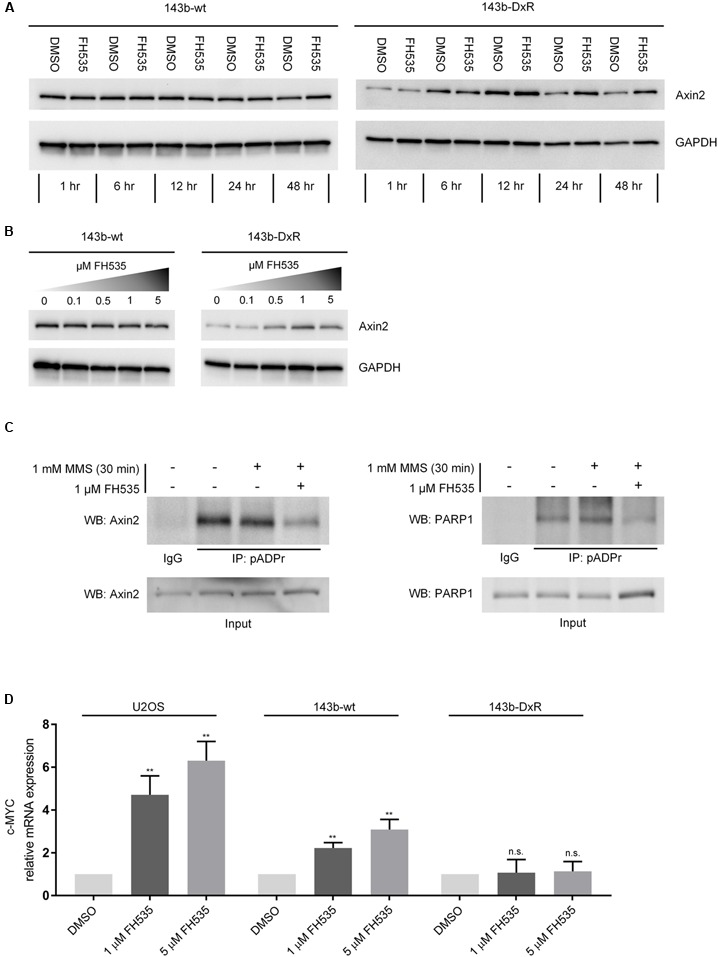
**FH535 inhibits PARylation by TNKS and PARP1. (A)** Axin2 protein expression over time during treatment with 1 μM FH535 in 143b-wt and 143b-DxR cell lines. **(B)** Axin2 protein expression in 143b-wt and 143b-DxR cell lines after 48 h treatment with increasing doses of FH535. **(C)** Immunoprecipitation for pADPr in the 143b-DxR cell line following treatment with 1 μM FH535 for 48 h. **(D)** Expression of c-MYC mRNA after 24 h treatment with FH535 in 143b-wt, U2OS, and 143b-DxR cell lines. (*n* = 3). Data information: in **(A–C)**, figures shown are representative images of triplicate experiments. **(D)** Data are presented as mean, ±SD. Statistical significance determined by one-way ANOVA with Tukey’s multiple comparison test, ^∗∗∗∗^ indicates *p* < 0.0001, “n.s.” indicates no statistically significant difference.

While several groups have clearly shown FH535 to inhibit canonical Wnt signaling via β-catenin, the molecular target of FH535 had yet to be identified (Bjorklund et al., 2014; Gedaly et al., 2014; Liu et al., 2014). Consistent with reports in other cell models, our study demonstrates FH535 inhibition of β-catenin transcriptional activity (Topflash reporter) (**Figure 3A**). In further support of β-catenin inhibition, we found that Axin2 mRNA transcript levels were inhibited by FH535 treatment at 24 and 16 h in the 143b-wt, 143b-DxR, and U2OS cell lines (**Figure 3B** and Supplementary Figure 2A). Treatment with FH535 resulted in stabilization of Axin2 protein, a β-catenin transcriptional target (**Figures 4A,B**). This observation was marked in the 143b-DxR cell line (**Figures 4A,B** right panels), while the 143b-wt cell line showed little change in Axin2 protein, corresponding to the decreased sensitivity to FH535 in the 143b-wt cells. Additionally, FH535 stimulated Axin2 accumulation in the U2OS cell line (Supplementary Figure 2C).

### PARylation of Axin2 Is Inhibited by FH535 in Osteosarcoma Cells

The inverse responses observed in Axin2 mRNA and protein suggested that Axin2 protein accumulation was a result of decreased degradation rather than increased production. The TNKS1/2 enzymes regulate Axin2 degradation by PARylation, a mechanism which was elucidated in Huang et al. (2009). Results from recently developed TNKS1/2 inhibitors have shown stabilization of Axin2 protein, decrease in Axin2 mRNA, and Wnt inhibitory activity, comparable to the observed effects of FH535 in OS (Bao et al., 2012; Quackenbush et al., 2016). Inhibitors of TNKS1/2 block Wnt signaling by reducing the PARylation of Axin2 by TNKS1/2. Thus, to test the ability of FH535 to inhibit TNKS1/2, we performed immunoprecipitation of PAR-modified proteins using a method similar to that described by Gagne et al. (2011). The results demonstrated that Axin2 is PAR-modified in OS cells, and FH535 significantly blocked PARylation of Axin2 (**Figure 4C** left panel).

### FH535 Blocks PARP1 Auto-PARylation and Upregulates c-MYC mRNA

We also assessed potential non-specific activity against total cellular PARylation, by co-immunoprecipitation for PAR-modified PARP1 following FH535 treatment. This experiment showed clear blockade of PARP1 auto-PARylation during FH535 treatment, in addition to blockade of Axin2 PARylation (**Figure 4C** right panel). Additionally, we found that c-MYC mRNA was strongly increased in the 143b-wt and U2OS cell lines following FH535 treatment for 24 h (**Figure 4D**) or 16 h (Supplementary Figure 2B). In contrast, treatment with IWR-1, a highly specific inhibitor of the TNKS1/2 enzymes, did not induce c-MYC mRNA expression (Supplementary Figure 3) (Chen et al., 2009; Lu et al., 2009; Narwal et al., 2012). This finding is added evidence of FH535 inhibition of PARP1 auto-PARylation, as unmodified PARP1 acts as a co-activator of c-MYC, in association with the transcription factor E2F-1 (Simbulan-Rosenthal et al., 2003). We further explored the possibility that FH535 may cause the observed blockage of Axin2 PAR-modification by inhibited expression of TNKS1 or TNKS2, and found no change in TNKS1 or TNKS2 mRNA expression following FH535 treatment (Supplementary Figure 4).

## Discussion

Outcomes among OS patients with local or metastatic disease have remained constant since the introduction of neoadjuvant chemotherapy (Isakoff et al., 2015). The regimen of methotrexate, Adriamycin (doxorubicin), and cisplatin has been established as the standard of care for OS. Recent trials testing the therapeutic value of combination therapies, such as interferon-α-2b or addition of ifosfamide and etoposide, have not reported significant improvements (Bielack et al., 2015; Marina et al., 2016). In Europe, mifamurtide has been approved for use in OS following a Children’s Oncology Group trial, although it has not yet found widespread acceptance, and is not approved in the United States (Chou et al., 2009; Ando et al., 2011). These findings highlight the need for the evaluation of molecular susceptibilities in OS, and the development of corresponding targeted therapies (Cleton-Jansen et al., 2009). The development and characterization of highly specific inhibitors of Wnt signaling provides new possibilities for treatment options in OS and other Wnt-dependent cancers. The present work demonstrates the cytotoxic effects of the small molecule FH535 in OS cells and elucidates the molecular mechanism by which FH535 inhibits Wnt signaling.

A number of studies have highlighted conferred dependency on Wnt in a variety of cancers, driven by mutations in APC or β-catenin (Fukushima et al., 2001; Filipe et al., 2009). Indeed, several reports have demonstrated pharmacologic inhibition or genetic silencing of TNKS1/2 to have strong tumor inhibiting effects in cellular and animal models of cancers (Casás-Selves et al., 2012; de la Roche et al., 2014). In contrast, APC and β-catenin driving mutations in OS are not widely reported, although Wnt dependence has been clearly shown in OS. However, the study by Stratford and colleagues reported OS susceptibility to tankyrase inhibition (Stratford et al., 2014). The findings in the Stratford study, together with the work reported here (**Figures 1**, **2**) suggest that TNKS1/2 directed therapies in OS and other cancers not known to contain frequent Wnt-driver mutations may prove to be a beneficial course of action. While many groups have reported Wnt-inhibition as a means to sensitize to various chemotherapy toxins, our data demonstrate that chemotherapy resistant cells may exhibit a more robust response to Wnt inhibition alone (**Figure 2**) (Dieudonné et al., 2012; Wickström et al., 2015; Zhang et al., 2016). Recent work has shown the upregulation of Wnt markers in response to DNA-damaging chemotherapies (Alakhova et al., 2013; Martins-Neves et al., 2016; Zheng et al., 2016). Thus, exposure to DNA-damaging molecules may select for a population of cells which depend on Wnt signaling. These reports may aid to explain the sensitivity of the doxorubicin-resistant cells to FH535 treatment.

The data in this report indicate that FH535 may target TNKS1/2 and PARP1, resulting in Wnt inhibition and reduction in OS cell survival. The TNKS1/2 enzymes positively regulate canonical Wnt signaling by facilitating the destruction of Axin2. TNKS1/2 inhibitors reduce PARylation of Axin2, and limit canonical Wnt signaling through β-catenin. Studies which have analyzed the affinity of TNKS1/2 inhibiting compounds have shown variable specificity for the PARP-family enzymes. XAV939, a TNKS1/2 inhibitor, is reported to cross-react with PARP1 with an IC_50_ of 0.11 μM and with PARP2 at 2.2 μM (Huang et al., 2009; Gunaydin et al., 2012). The majority of characterized TNKS1/2 inhibitors have been shown to have affinity for either the nicotinamide subsite of TNKS1 or TNKS2, the adenosine subsite, or both subsites of the TNKS1/2 enzymes (Lehtiö et al., 2008). Due to the high degree of conservation of the nicotinamide subsite between PARP family members, it has been hypothesized that the adenosine subsite is more desirable for TNKS1/2-specific targeting (Gunaydin et al., 2012). Indeed, adenosine-site directed inhibition has been achieved by IWR-1 and G007-LK; as these small molecules exhibit decreased cross-reactivity with other PARPs (Lu et al., 2009; Voronkov et al., 2013). The data within this study (**Figure 4**) suggest that FH535 may be grouped with other small molecules which have been shown to inhibit both PARP1 and TNKS1/2. Detailed examination of the structure-activity relationship of FH535 in the context of PARP1 and TNKS1/2 may facilitate increased specificity through rational modification of the molecule. Still, secondary inhibition of PARP activity may be therapeutically beneficial in OS, particularly when combined with targeting of Wnt signaling, as demonstrated by *in vitro* data in this study.

While this study reports the mechanism of FH535 activity in OS cell lines, and presents *in vitro* data that demonstrate toxicity to OS cells, further work will be required to show the broad application and *in vivo* potential of the drug. The doxorubicin resistant model developed in this study is an example of one type of chemotherapy resistance, while other mechanisms may also be involved in regulating the sensitivity of OS to various chemotherapy regimens. Thus, while OS dependency on Wnt has been widely reported *in vitro*, as well as in animal and human samples of OS, our model may represent a single subset of human OS cells which have developed resistance to currently used chemotherapeutic agents (Zhao et al., 2015; Martins-Neves et al., 2016). Additional work will also be required to determine the efficacy, stability, and pharmacokinetics of FH535 in animal models of OS, as these properties have been highly variable in other molecules targeting the TNKS1/2 enzymes (Lu et al., 2009).

In sum, this study details the susceptibility of OS, including a model of chemotherapy resistant OS, to growth inhibition with the small molecule FH535. Additionally, the study clarifies the mechanism by which FH535 inhibits Wnt signaling, and reports the inhibition of PARP1 auto-modification in addition to TNKS1/2 blockade. Our results suggest that Wnt-targeting chemotherapeutics, such as FH535, may be promising candidates for treatment of OS.

## Author Contributions

CG: wrote the manuscript, analyzed data, performed experiments, and planned the study. TM: performed experiments and analyzed data. KS: performed experiments. AM: planned the study and interpreted data. MY: planned the study and interpreted data.

## Conflict of Interest Statement

The authors declare that the research was conducted in the absence of any commercial or financial relationships that could be construed as a potential conflict of interest.
