# Supplementary material for: FH535 Suppresses Osteosarcoma Growth In Vitro and Inhibits Wnt Signaling through Tankyrases
Source: Front Pharmacol. 2017 May 23;8:285. doi: 10.3389/fphar.2017.00285 (PMC5440578; doi:10.3389/fphar.2017.00285)

Supplementary Material

FH535 suppresses osteosarcoma growth *in vitro* and inhibits Wnt signaling through tankyrases

Carl T. Gustafson[1], Tewodros Mamo[1], Kristen L. Shogren[2,3], Avudaiappan Maran[2,3], Michael J. Yaszemski[1,2,3]

Correspondence: Carl T. Gustafson, gustafson.carl@mayo.edu

Table of Contents:

Supplementary Figure 1 – Effects of FH535 on cell survival.

Supplementary Figure 2 – Expression of Wnt target genes regulated by FH535

Supplementary Figure 3 – Tankyrase1/2 mRNA expression after FH535 treatment

Supplementary Figure 4 – c-MYC expression regulated by IWR-1 treatment

Supplementary Figure 1:

(A) Live / Dead staining of OS cell lines after 48 hour treatment with FH535 in U2OS, K7M2, and 143b cell lines.

(B) Colony forming ability of 143b-wt cell line during treatment with FH535.

Data information: in A, figures shown are representative images of triplicate experiments. B, data are presented as mean, +/- s.d. (n = 3).

A

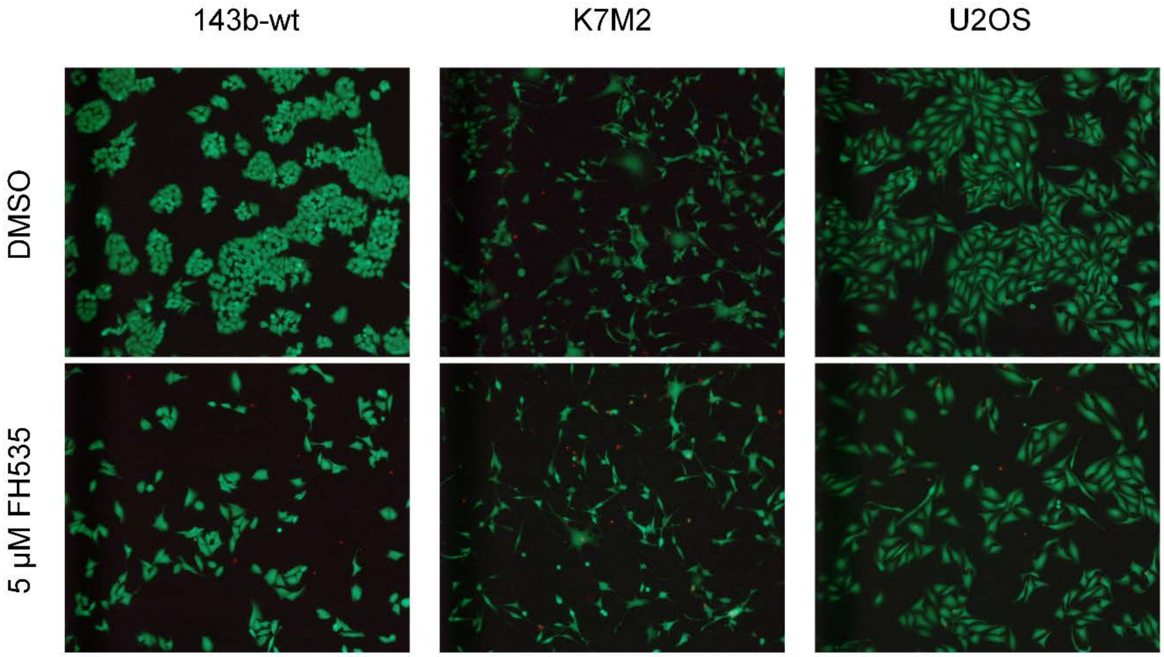

B

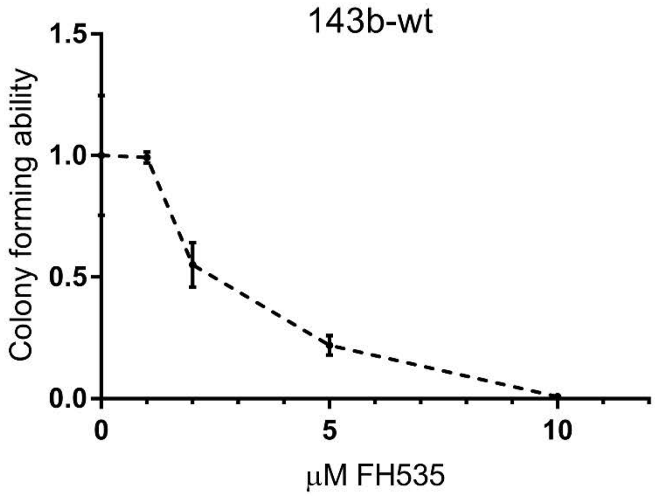

Supplementary Figure 2:

(A) Quantification of Axin2 mRNA in the U2OS, 143b-wt, and 143b-DxR cell lines after 16 hr treatment with FH535.

(B) Quantification of c-MYC mRNA in the U2OS, 143b-wt, and 143b-DxR cell lines after 16 hr treatment with FH535.

(C) Axin2 protein regulation following 48 hr treatment with FH535 in U2OS cell line.

Data information: in A-B, data are presented as mean, +/- s.d. (n = 3). In C, figure shown is a representative image of triplicate experiments. Statistical significance determined by one-way ANOVA corrected for multiple comparisons, * indicates p<0.05, ** indicates p<0.01, **** indicates p<0.0001, “n.s.” indicates no statistically significant difference.

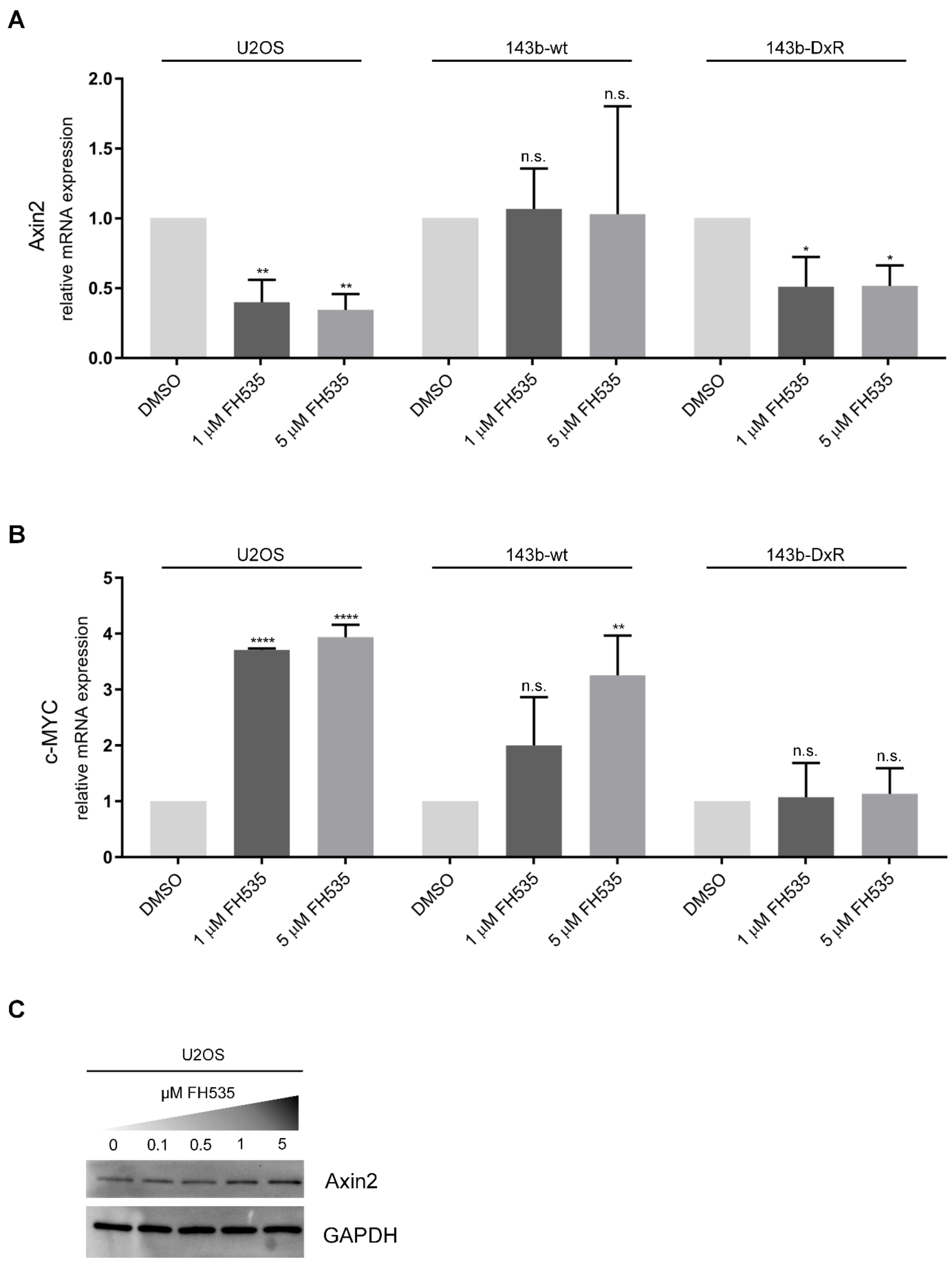
A
U2OS
143b-wt
143b-DxR
Axin2
relative mRNA expression
2.0
1.5
1.0
0.5
0.0
**
**
n.s.
n.s.
*
*
DMSO
1 μM FH535
5 μM FH535
B
U2OS
143b-wt
143b-DxR
c-MYC
relative mRNA expression
5
4
3
2
1
0
****
****
n.s.
**
n.s.
n.s.
DMSO
1 μM FH535
5 μM FH535
C
U2OS
μM FH535
0
0.1
0.5
1
5
Axin2
GAPDH

Supplementary Figure 3:

TNKS1 and TNKS2 mRNA expression in 143b-DxR cell line following 48 hour treatment with FH535.

Data information: data are presented as mean, +/- s.d. (n = 3). Statistical significance determined by unpaired t-test with Welch's correction, "n.s." indicates no statistically significant difference.

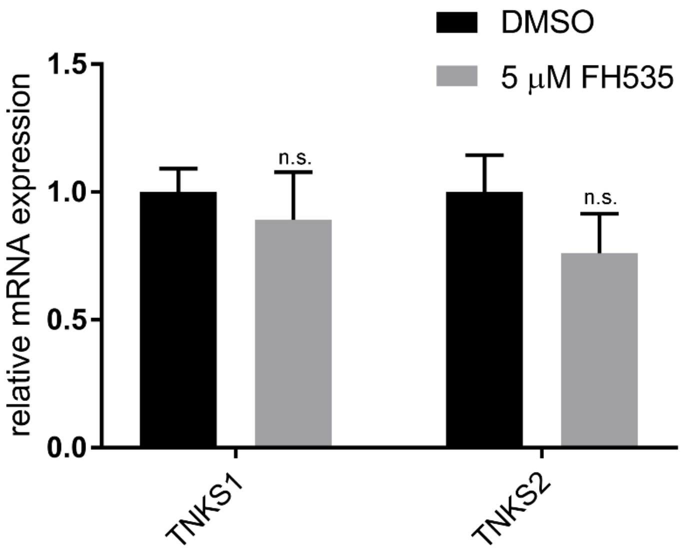

Supplementary Figure 4:

Quantification of c-MYC mRNA in the U2OS, 143b-wt, and 143b-DxR cell lines after 24 hr treatment with IWR-1.

Data information: data are presented as mean, +/- s.d. (n = 3). Statistical significance determined by one-way ANOVA corrected for multiple comparisons, ** indicates $p<0.01$, *** indicates $p<0.001$, "n.s." indicates no statistically significant difference.

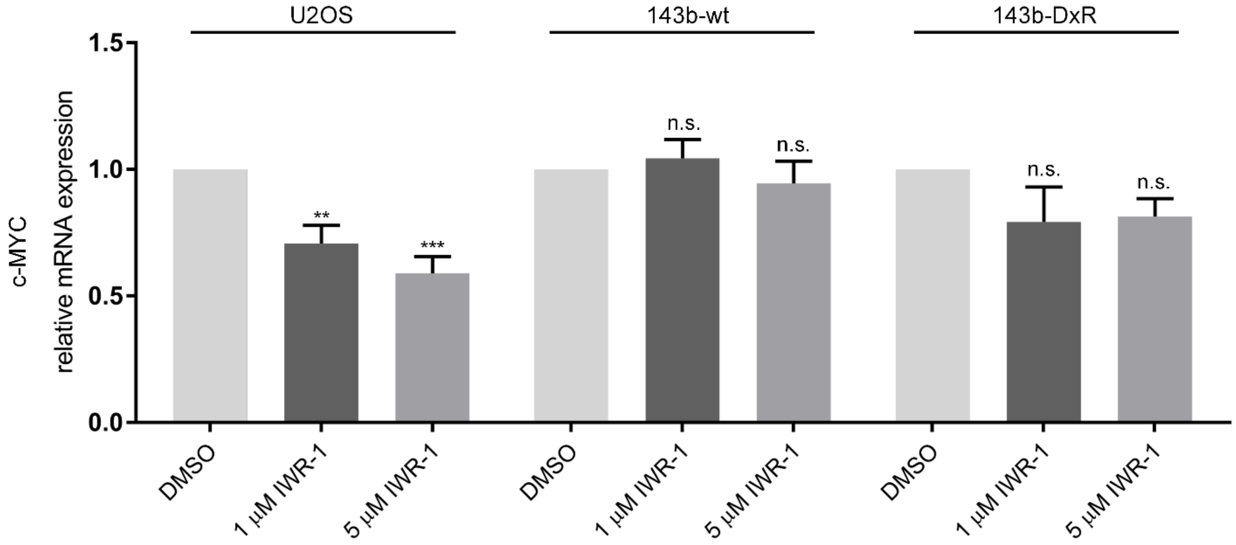

Supplement: Supplementary file 1 [file Data_Sheet_1.PDF]
